# Factors Associated with Autopsy Consent in Pediatric Oncology: A 10-Year Review

**DOI:** 10.3390/curroncol33050297

**Published:** 2026-05-20

**Authors:** Meaghann S. Weaver, Jia Liang, Rachel Jalfon, Yimei Li, Abagail D. Cohen, Liza-Marie Johnson

**Affiliations:** 1 Bioethics Program, St. Jude Children’s Research Hospital, Memphis, TN 38105, USA; 2 Division of Palliative Care, Department of Oncology, St. Jude Children’s Research Hospital, Memphis, TN 38105, USA; 3 Department of Biostatistics, St. Jude Children’s Research Hospital, Memphis, TN 38105, USA

**Keywords:** autopsy, pediatric oncology, informed consent, decedent study

## Abstract

Purpose: Autopsy remains an important diagnostic and research modality in pediatric oncology. This study examined demographic and clinical factors associated with parental acceptance or decline of autopsy in childhood cancer. Patients and Methods: This study was a retrospective chart review of autopsy consent acceptance or decline patterns between 2007 and 2017 for inpatient pediatric oncology deaths in a large single-site oncology hospital. Demographic factors (age, race, gender), diagnostic factors (primary cancer, transplant history, and neurologic status 24 h prior to death), interventions (intensive care unit location, dialysis, ventilator, chemotherapy, medically administered nutrition), and code status in the 24 h prior to death were obtained. Analysis included descriptive and statistical correlations. Results: Among 344 inpatient decedents, 34% of families consented to autopsy. There was a difference in consent rate according to race (*p* = 0.015). Diagnosis, transplant status, age, and neurologic status showed no association. Use of dialysis (*p* < 0.001), ventilation (*p* < 0.001), and intensive care unit (ICU) location (*p* < 0.001) correlated with higher consent rates. Chemotherapy and assisted nutrition were not associated with decisions. Presence of a Do Not Resuscitate (DNR) order predicted lower consent (*p* < 0.001), while receipt of cardiopulmonary resuscitation (CPR) at death predicted higher consent (*p* < 0.001). Conclusion: One-third of families of inpatient pediatric oncology decedents with cancer agreed to autopsy. Demographic and diagnostic factors were not universally strong predictors, underscoring the personal nature of autopsy decisions. Further research should include multisite prospective designs and direct engagement with bereaved families.

## Simple Summary

Families of children with cancer consented to autopsy in approximately one-third of cases, with decisions influenced more by circumstances surrounding end-of-life care than by demographic or diagnostic factors. Higher consent was associated with ICU care, life-sustaining treatments, and CPR at death, highlighting the need for unbiased and consistent approaches to autopsy inquiry and deeper understanding of family perspectives.

## 1. Background

Autopsies are a diagnostic and research tool to advance pediatric oncology knowledge. Autopsies retain importance in pediatric oncology due to the biological complexity of childhood cancers, the frequent use of intensive multimodal therapies, and the limited availability of tumor tissue before death in certain diagnoses. Despite progress regarding imaging, molecular diagnosis, and less invasive biopsy methods, autopsies hold a unique place [1]. Autopsies provide a thorough postmortem evaluation of tumor burden, cancer spread, and treatment-related side effects, along with other potential causes of death that may have not been understood before death [1,2,3]. Several historic studies conducted among pediatric and adult oncology populations demonstrate the ongoing value of autopsy with the documentation of significant findings, including major diagnostic discrepancies that apply to approximately 10–30% of cases even with the advanced medical knowledge of the present day [4,5,6,7]. Among pediatric oncology patients, the findings of a postmortem examination documented newly discovered disease spread, infectious/inflammatory processes, and organ insights [8,9]. These findings underscore the potential continued relevance of autopsy for diagnostic accuracy and ongoing research efforts in oncology care.

Beyond a diagnostic role, autopsies have become largely valuable for childhood cancer research, particularly in certain pediatric oncology diagnoses in which tissue scarcity limits biologic discovery. Postmortem tissue acquisition allows for increased sampling of primary tumors and metastatic sites, many of which are inaccessible during life, enabling studies of tumor evolution, clonal heterogeneity, and mechanisms of therapeutic resistance [10,11,12]. Autopsy-derived tissue has been instrumental in advancing understanding of aggressive and lethal pediatric malignancies such as diffuse midline glioma, neuroblastoma, and sarcomas, and has supported the development of preclinical models and translational research initiatives [13,14,15]. Integrated autopsy programs further demonstrate the feasibility and scientific value of integrating autopsy into cancer research infrastructures [10,16].

Even with the established diagnostic and research value, autopsy utilization has declined over recent decades [2,3]. Pediatric hospital autopsy rates in high-income countries have fallen from historical levels exceeding 30–40% to well below 10%, with many institutions reporting rates under 5% [3,17,18]. Pediatric oncology-specific data are limited, but evidence suggests similarly low uptake, even in tertiary care centers managing medically complex children [19]. In adult oncology, where more comprehensive data exist, autopsy rates are frequently reported at less than 5%, with some academic centers documenting rates below 2%, despite continued identification of unexpected and clinically meaningful findings [7,20,21]. In contrast, higher autopsy rates persist in select non-oncologic pediatric deaths, such as sudden unexplained deaths and certain intensive care unit fatalities, where standardized institutional and medical–legal processes are more firmly established [3,22].

The factors for persistently low autopsy rates in pediatric oncology are complex and multifaceted. One facet may be the concurrent advances in pathological, genetic, and imaging technologies. Many studies identify physician reluctance to introduce discussions of autopsy as a possible impediment. Clinicians report discomfort raising the topic during periods of intense family grief, fear of exacerbating distress or harming therapeutic relationships, and uncertainty regarding appropriate timing and communication strategies [23,24,25]. These concerns are often heightened by limited formal training in autopsy consent and the lack of a consistent approach to families regarding the postmortem examination [26]. In our setting during the study period, all families were approached prior to or immediately after death as part of standardized end-of-life processes regarding autopsy consent.

At our institution, autopsy findings are then communicated to families through a structured but flexible process. Preliminary results, when available, may be shared by the primary clinical team or pathology service within several weeks of the autopsy, particularly if findings have immediate clinical relevance or implications for family understanding. Final autopsy reports are typically completed within approximately 6–12 weeks, depending on the complexity of the case and the need for additional studies. Families are generally offered the opportunity to review results through a follow-up discussion, most often conducted by the primary clinical team in collaboration with pathology and, when appropriate, palliative care or bereavement services. These conversations may occur in person or by telephone, based on family preference and logistical considerations. During these discussions, clinicians aim to explain findings in clear, non-technical language, contextualize results in relation to the clinical course, and address questions or concerns. When relevant, implications for heritable conditions or risks to other family members are also discussed, with referral for additional counseling as needed. Despite these general practices, communication processes are not fully standardized and may vary by clinical team and circumstance.

Consistent and compassionate conversations with families about autopsies can be a means to foster equity in access to information obtained by autopsies, to foster truly shared decision making, and to honor family preferences. Informed consent conversations about autopsies should be a means for families to have equal opportunity to pursue or decline an action based on their ability to weigh the potential value or meaning. However, little is known about autopsy consent patterns in childhood cancer decedent cohorts as compared to adult populations. Identification of the demographic, clinical, and situational factors associated with autopsy consent is vital to clarify rates of autopsy agreement or decline. Insight into patterns of autopsy consent can help inform conversational approaches and improve family access to postmortem diagnostic and research options in pediatric cancer. The objective of this study was to quantify the autopsy consent accept or decline rate among all inpatient decedent oncology patients and to correlate this with demographic and clinical characteristics.

## 2. Materials and Methods

### 2.1. Population

All pediatric oncology patients who died within any inpatient location at our institution between 2007 and 2017 were included. These years were selected to avoid the influence of the COVID-19 pandemic, to include a full decade, and due to the use of a unified electronic health record for consistent data reporting in that timeframe. During the study years, a question regarding family autopsy consent agreement or decline was standardized in the medical record as a required item for completion of the death summary. The physician caring for the patient at the time of death was expected to verify whether consent had been obtained/discussed prior. If not, the physician was responsible to ask the family about autopsy consent as part of end-of-life care documentation. This retrospective chart review study was determined to be exempt from full institutional review board due to the decedent-only cohort.

### 2.2. Data Extraction

General demographics (age, gender, race, ethnicity, religion, and decision maker) and clinical information (primary cancer, transplant history, and neurologic status 24 h prior to death) were recorded. Specific interventions 24 h prior to terminal event were documented such as: intensive care unit (ICU) location, dialysis, ventilator, cure-directed chemotherapy, and medically administered nutrition and hydration (MANH). Documented code status and cardiopulmonary resuscitation (CPR) events at time of death were noted. Since all families are asked about an autopsy, the decedent chart was reviewed for autopsy consent agreement or decline. The study team collected data from electronic health records.

### 2.3. Statistical Analysis

Chi-squared tests were used to examine the association between two categorical variables, and Fisher’s exact test was used when there was a sub-category containing a count of less than five. The Wilcoxon rank sum test was used to examine the distributional difference in a continuous variable between two categories. Logistic regression was used to further examine the association between the decision on autopsy and last-day clinical interventions, while adjusting for age at death and primary diagnosis.

All analyses were performed at a 95% significance level, and all analyses were done using R 4.5.1 (R Foundation).

## 3. Results

A total of 344 decedent children with cancer were included in the study. Demographics are provided in Table 1. Approximately one-third (34%) of families consented to autopsy. There was a lower average age for the 116 children whose family consented to autopsy (mean 9.84 years, SD 7.11 years) as compared to the 228 who declined (mean 11.3 years, SD 6.76 years), but due to large variations in age, there was not a statistically significant difference by age (*p* = 0.0724). There was a difference in consent rate according to race (Table 2). Families of Asian and Black children consented to autopsy at a lower rate than White children or other racial groups (*p* = 0.015). As shown in Table 3, self-identification of the family as being in a known religion other than Christianity was also associated with autopsy decline (*p* = 0.0395).

Receipt of curative chemotherapy and medically administered nutrition in the 24 h prior to death were not associated with autopsy decisions. Use of intensive interventions such as dialysis (*p* < 0.001), ventilation (*p* < 0.001), and ICU location (*p* < 0.001) correlated with higher autopsy consent rates (Table 4).

Notably, patients with a substantial level of care escalation in the final day of life such as a transfer to the ICU on the final day of life (*p* = 0.0505) or intubated on the final day of life (*p* = 0.0586) were more likely to undergo autopsy. Receipt of CPR at death predicted higher likelihood of consent to autopsy (*p* < 0.001). Patients with autopsy had significantly higher rates of having undergone CPR at the time of the terminal event (61.5%) compared to those who did not undergo an autopsy (38.5%). The presence of a Do Not Resuscitate (DNR) order predicted lower consent to autopsy (*p* < 0.001).

## 4. Discussion

In this retrospective, single-site study of inpatient childhood cancer decedents, approximately one-third of families consented to autopsy. This rate is consistent with previously published pediatric autopsy rates [27,28,29]. Diagnostic factors such as cancer type, transplant status, and neurologic status were not universally strong predictors of autopsy agreement, underscoring the personal nature of these decisions and the need to avoid bias in offering autopsy based on diagnosis alone [7]. Associations with ICU care, more aggressive life-sustaining treatments in the final day of life, and receipt of CPR at time of death highlight contextual factors that may influence autopsy consent decisions in pediatric oncology.

The observation that autopsy uptake did not differ according to oncologic diagnosis is somewhat unexpected given the heterogeneity typically observed in cancer trajectories, prognostic clarity, and clinician–family decision making dynamics across disease types. Variation in disease course (such as the relative predictability of decline in certain solid tumors versus the more fluctuating trajectories seen in hematologic malignancies) can influence end-of-life practices, including the perceived utility of postmortem examinations. Therefore, one might reasonably hypothesize that diagnostic category would shape attitudes toward autopsy, either by clinicians seeking diagnostic clarification or by families motivated by uncertainty or unmet informational needs. Several possible explanations may account for the lack of observed variation. It is plausible that systemic and institutional factors exert a stronger influence on autopsy uptake than disease-specific characteristics. These may include hospital policies, availability of pathology services, clinician comfort with requesting autopsy, and broader cultural or regional norms surrounding postmortem examination. In such contexts, decision making may be standardized or routinized, thereby attenuating any differences that might otherwise arise from diagnostic heterogeneity. Additionally, advances in diagnostic imaging, molecular testing, and precision oncology may have reduced perceived uncertainty across many cancer types. As diagnostic confidence improves, clinicians and families may feel less need for autopsy to resolve outstanding clinical questions, leading to uniformly low uptake regardless of the underlying malignancy. This effect could be particularly salient if the clinical cause of death is viewed as well-characterized, even in cases with complex comorbidity profiles. Notably, the decision to pursue autopsy may be influenced more by communication processes and relational dynamics than by clinical variables alone. Factors such as the timing and framing of autopsy discussions, clinician attitudes, and family preferences may overshadow diagnostic considerations. If clinicians are not systematically differentiating their approach based on cancer type, or if families are not informed of potential diagnosis-specific benefits, uptake may appear homogeneous across groups. Taken together, it is important to consider that the absence of a detectable difference does not necessarily imply true equivalence. Statistical power, sample size, and categorization of oncologic diagnoses may limit the ability to identify more nuanced patterns. Aggregation of diverse malignancies into broad categories could obscure subtype-specific trends, particularly if certain cancers (e.g., those with higher rates of diagnostic ambiguity or unexpected complications) are underrepresented. Taken together, the lack of difference in autopsy by diagnoses suggests that autopsy utilization may be driven not solely by clinical specifics of oncologic diagnosis but also by structural, communicative, and cultural factors within healthcare systems. Future work could explore these dimensions in greater detail, including qualitative assessments of clinician and family perspectives, to better understand the determinants of autopsy decision making in oncology.

The cadence of ongoing cure intent chemotherapy and medically administered nutrition was not significantly associated with autopsy consent. In contrast, indicators of intent chemotherapy and medically administered nutrition were not significantly associated with autopsy consent. Escalation to these interventions on the final day of life showed a particularly strong association with consent, suggesting that the rapidity or severity of clinical decline may influence families’ intensity of medical care—such as ICU location in the final 24 h and receipt of interventions including dialysis and mechanical ventilation—which was associated with higher autopsy consent rates. Escalation to these interventions on the final day of life showed a particularly strong association with consent, suggesting that the rapidity or severity of clinical decline may influence families’ decision making. Undergoing cardiopulmonary resuscitation at the time of death was also predictive of autopsy, with markedly higher CPR rates among decedents who had an autopsy performed. Autopsy in these contexts may reflect a desire for greater understanding of the proximate cause of death, especially when the terminal event is unexpected, acute, or medically complex.

The study did not specifically inquire why respondents for the decedents failed to provide autopsy consent. Reasons for refusal cited in the existing autopsy literature vary from assumptions that the cause of death is already known, lack of perception that the autopsy would yield new or beneficial information, belief about maintaining the intactness of a physical body after death, or any combination of cultural, religious, and even emotional factors [24,30,31]. Prior research has shown that families worry that an autopsy would delay funeral arrangements, prevent viewing of the body as part of a funeral, or other postmortem logistics [32,33]. Clarity regarding the tangible timelines and processes can help clarify logistical misconceptions that may compel decline.

Race was significantly associated with autopsy consent in this cohort. Prior research in pediatric and adult populations has demonstrated varied patterns of autopsy utilization across racial and ethnic groups, with some studies reporting higher nonforensic autopsy consent among Black families compared with White families, while others describe lower rates depending on clinical context, geography, and historical factors [34]. Previous work has also noted reduced autopsy uptake among certain Asian populations, including geographically defined cohorts of parents of stillborn infants, where cultural, spiritual, and communication factors may influence decision making [35]. Epidemiological studies from several Asian countries similarly report autopsy rates below 3%, though these reflect specific health systems, legal frameworks, and cultural norms rather than generalizable group characteristics [36,37,38,39,40].

Racial or ethnic associations with autopsy consent should be interpreted with humility and caution, recognizing that race is a social construct and does not represent uniform beliefs or behaviors. Observed differences based on race should be interpreted within the context of structural and systemic factors rather than as inherent or biologically determined characteristics. Race, as captured in this study, is a social construct that may reflect differences in access to information, prior experiences with healthcare institutions, communication practices, and levels of trust shaped by historical and ongoing inequities. Variation in autopsy consent may reflect historical experiences, structural inequities, differences in how autopsy is offered or explained, and disparities in trust toward health systems [41]. Variability in how autopsy is introduced, explained, and discussed (including differences in clarity, completeness, and cultural responsiveness) may realistically contribute to observed patterns in consent. These factors underscore the importance of avoiding reductionist interpretations and instead highlight the need for equitable communication practices, standardized offering processes, and efforts to build trust. Future work should more directly examine these structural and interpersonal determinants to better understand and address disparities in autopsy consent.

Clinicians have an ethical responsibility to ensure that autopsy is offered equitably, communicated in a culturally sensitive manner, and presented with accurate information about available options and potential value. Autopsies should be offered equitably within relevant pediatric cancer diagnoses so that there are fair opportunities for research representation and knowledge exchange. Each family should be supported to make an autonomous, values-aligned decision, without assumptions based on race, ethnicity, or religion [24]. Emerging evidence suggests that minimally invasive autopsy approaches may increase uptake among some groups in adult care settings [42], potentially offering additional avenues to support equitable aligned decisions, without assumptions based on race, ethnicity, or religion.

The clinical care team should consider engaging cultural liaisons, community advisors, spiritual leaders, and chaplaincy services to support culturally informed discussions about autopsy. These colleagues can provide valuable contextual insight into general belief systems, practices surrounding death, and potential sources of concern, thereby helping clinicians approach conversations with greater awareness and sensitivity. Palliative care and psychosocial colleagues add specialized communication expertise, enhancing sensitivity, emotional attunement, and support during autopsy discussions. Such partnerships can enhance clinician confidence by offering guidance on how to frame discussions in ways that are respectful and aligned with broadly held values, while still preserving the principle of individualized and family-centered care. Importantly, these resources should be used to inform (not replace) direct engagement with families, ensuring that decisions remain grounded in each family’s unique perspectives rather than cultural stereotypes or generalized assumptions.

Physician avoidance, most often out of sensitivity about approaching grieving families, appears to be a main barrier to obtaining autopsy consent cited in the existing literature [5]. Importantly, there is emerging data to suggest that clinician reticence may not accurately mirror parental desire. Treatment teams may exhibit varying levels of comfort when engaging in discussions about autopsy. For instance, neuro-oncology teams with a high population of patients with Diffuse Intrinsic Pontine Glioma (DIPG) or Atypical Teratoid Rhabdoid Tumor (ATRT), or other clinicians caring specifically for diagnoses cohorts with high mortality rates, could be more accustomed to and therefore more at ease initiating such conversations compared to teams specializing in diagnoses with anticipated curability. Similarly, individual clinicians may differ in their personal and professional comfort with autopsy-related discussions, with some demonstrating greater ease and willingness to engage in these conversations, while others may experience varying degrees of discomfort or reluctance.

Clinicians’ confidence in offering autopsy consent can be enhanced through structured communication training and approaches that emphasize transparency, normalization, and cultural humility. Rather than positioning autopsy as an exceptional or uncomfortable request, it is recommended that clinicians frame it as a routine component of comprehensive end-of-life care, with potential benefits for diagnostic clarification, quality improvement, and, in some cases, family understanding. Communication frameworks such as Ask–Tell–Ask can provide a consistent structure for these discussions, helping clinicians introduce the topic sensitively while responding to emotional cues. Importantly, culturally sensitive practice should not rely on assumptions based on a patient’s or family’s background; instead, it should center on eliciting individual values, beliefs, and preferences. Practical strategies include language that supports cultural sensitivity. A statement such as “We recommend an autopsy” may be experienced as declarative or directive. An exemplar approach may be to phrase this same sentiment with an invitation for reflection: “One option we offer families is an autopsy to better understand what happened. I’d like to explain it, and then I’d like to welcome your reflections or thoughts.” Open-ended questions about spiritual practices, concerns regarding the body, or expectations after death allow clinicians to tailor the conversation respectfully without stereotyping. While awareness of common cultural themes (such as concerns about bodily integrity or the timing of burial) can inform these discussions, best practice is to adapt the approach rather than the offer itself. In other words, autopsy should be offered equitably to all families, with modifications made responsively based on expressed values rather than presumed norms.

The American Academy of Pediatrics Guidance for End-of-Life Care recommends that all families should have access to hearing about the potential benefits of full or limited autopsy [42]. Almost all (93%) bereaved parents of children with cancer endorse that they would have consented to research autopsies if offered, yet only half were approached [43]. A prospective multicenter study of 33 bereaved parents of children with diffuse intrinsic pontine glioma who consented for participation in research autopsies reported no regrets [44]. Some bereaved parents may view autopsy as a means to learn more specifically about their child, to contribute to medical progress, and to advance science altruistically while honoring their child’s legacy [43,45].

Research into autopsy consent in the setting of pediatric oncology has demonstrated that parents are more likely to consent to autopsy if the process is handled uniformly, without bias, and is conducted through well-established institutional processes [43,45]. Some settings offer autopsy to every child with cancer around the time of death as a standard of end-of-life care. It is important to recognize that the objective of the autopsy often is research without a sure direct clinical benefit for the patient. Pediatric oncologists are aware of the challenge and the additional stress to patients and families when seeking consent for clinical trial participation when parents are faced with a life-threatening diagnosis [28]. A significantly higher degree of sensitivity to continue investigation for research purposes at the time of a child’s death is required. Standardization in the autopsy approach based on diagnoses and communication training may improve consent practices.

Several pediatric centers lack standardized processes to help guide the eligibility of autopsy candidates, identify the staff responsible for starting discussions about autopsy, and assist with the coordination of the pathology team [46]. Difficulty related to logistics (such as delays in the schedule of morgue transfer or funeral), the 24/7 availability of pathology expertise for pediatric patients, and lack of a clinical research autopsy service might dissuade clinicians from performing the procedure despite the possibility of parental receptiveness [25]. Establishment of a dedicated team, such as a Decedent Affairs point of contact, to serve as a direct link between pathology and families has been shown to increase autopsy consent rates [47]. Successful implementation of autopsy programs requires communication training for clinical care teams, creating established and consistent consent approach practices, and establishing infrastructure paired with functional standard operating procedures specific to diagnoses with the greatest autopsy relevance [31,48,49,50].

The data regarding autopsy purpose and practices are notably historic (as evidenced by the years of publication included in this manuscript’s references despite extensive search terms with medical librarian assistance). The decline in population uptake of autopsy consent and the aged nature of the existing data raises consideration as to whether autopsies may have a shifting relevance today. The role of autopsy today may be different than it once was (and perhaps less impactful, scientifically, socially, or otherwise). This shifting state of autopsy benefit for certain childhood cancer diagnoses could be an under-explored reason for clinicians’ reluctance to approach families about autopsy.

From a scientific perspective, autopsies also offer unique insights into disease biology, particularly in complex or rare conditions, by enabling comprehensive tissue-based evaluation that is often not feasible during life. These findings may contribute to hypothesis generation and a deeper understanding of pathophysiology. In this study, however, we did not have access to detailed case-level data necessary to systematically evaluate the contribution of autopsy to diagnostic refinement, identification of complications, or novel biological insights, nor to assess concordance between antemortem and postmortem findings. This represents an important limitation. Future research should incorporate prospective, standardized assessment of diagnostic concordance and clinical impact to better quantify the utility of autopsy in contemporary practice. Futuristically, the essential role for autopsies may be shifting in pediatric oncology practice as diagnostic technologies advance. This is particularly true in the case of leukemia, the most common childhood cancer, where access to tumor tissue at diagnosis as well as at relapse is easily accomplished via blood and bone marrow. With the advancing state of the art pathological methods, including exome and transcriptomic sequencing, autopsy results may yield less diagnostic clarity in the current and future times than in the past. Additionally, future advances in imaging technology may eventually preclude the need for anatomical evidence of disease spread obtained by autopsy. The situation is admittedly different with specific rare cancers, with diffuse, high-grade midline gliomas being the best appreciated example. However, biopsies are now more regularly entertained to establish the diagnosis and presence of prognostic biomarkers, and limited autopsy techniques to aid tumor specimen procurement for biological investigations have become an accepted option and widely recommended by patient advocates [21]. Future work should explore the frequency of more accurate diagnostic information made available by autopsy series in the setting of advanced technologies during a child’s diagnosis and treatment course [5,6,7].

Limitations of this study include that the cohort included only inpatient deaths in a single cancer research referral site. The study did not track patient access to or exposure to charitable organizations, tumor donation studies, or palliative and psychosocial care, thereby limiting our ability to assess correlations with autopsy uptake patterns. The unique demographics, research and care practice models, and resources in our setting may limit generalizability to other health systems. We only included inpatients and therefore our findings are exclusive of patients who may have died at home, which introduces outcome-dependent sampling bias since there may be important demographic or clinical differences in the location of death. The retrospective observational design limited our ability to establish causal relationships. Specifically, there was no follow-up question or data collection point to capture the underlying reasons behind “why” parents accepted or declined autopsies. Since the timing of this study, we have implemented “reason for decline” as a standard question in our autopsy communication documentation template as a practice improvement. The absence of standardized or documented communication practices introduces the possibility of information bias, as differences in clinician approach, completeness of information provided, or family understanding may have affected observed consent patterns. Important next steps would be to engage the perspectives of parents regarding value-based autopsy consent timing, communication processes and topics, and cultural consideration. Autopsy (and autopsy consent) practices might change over time, either generally and/or in specific subgroups. Future research incorporating multisite cohorts, prospective designs, and direct engagement with bereaved families will be important to deepen understanding and support equitable, values-aligned autopsy practices in pediatric oncology.

## 5. Conclusions

Autopsy remains a diagnostic and research tool in pediatric oncology. Autopsy consent was associated with clinical circumstances surrounding the terminal event, including care in the ICU, receipt of intensive life-sustaining interventions, and undergoing cardiopulmonary resuscitation at the time of death. These contextual factors suggest that families may seek answers or clarification in the setting of unexpected or escalated medical events. Additional research is warranted to prospectively explore the motivations and meaning that parents place on autopsy decisions. Demographic associations, including those observed with race and religion, warrant thoughtful interpretation and emphasize the need for clinicians to approach families with equitable, culturally respectful autopsy discussions. Diagnostic characteristics such as cancer type, transplant history, neurologic status, and use of curative intent therapies were not strong predictors of autopsy decisions, underscoring that these choices are deeply personal and value based. We therefore recommend approaching families about autopsy options with humility and inclusivity. Strengthening clinician communication skills, establishing standardized institutional processes for pediatric oncology diagnoses with most relevance for autopsy benefit, and ensuring timely coordination with pathology teams may enhance both the equity and quality of autopsy practices. Improving autopsy processes in pediatric oncology has the potential to advance diagnostic accuracy, expand research tissue availability, and support families through compassionate, informed end-of-life care.

## Figures and Tables

**Table 1:** Decedent demographics.

Gender	Male	205 (59.6%)
	Female	139 (40.4%)
Race	White	223 (67.5%)
	Black/African American	85 (24.6%)
	Other	14 (4.1%)
	Asian	6 (1.7%)
	Unknown	1 (0.3%)
Ethnicity	Non-Hispanic	267 (77.6%)
	Hispanic/Latino	70 (20.3%)
	Unknown	7 (2%)
Religion	Christian	273 (79.4%)
	Unknown/Unaffiliated	60 (17.4%)
	Muslim	6 (1.7%)
	Hindu	2 (0.6%)
	Other	2 (0.6%)
	Jewish	1 (0.3%)
Decision Maker	Both Parents	190 (55.2%)
	Mom	84 (24.4%)
	Self with Parent Actively (loss of capacity)	32 (9.3%)
	Other	16 (4.7%)
	Dad	10 (2.9%)
	Self	12 (3.5%)

**Table 2:** Association between autopsy and race.

	Asian (N = 6)	Black (N = 85)	Other (N = 29)	Unknown (N = 1)	White (N = 223)	*p*-Value
Autopsy						
No	6 (100%)	65 (76.5%)	17 (58.6%)	0 (0%)	140 (62.8%)	0.015
Yes	0 (0%)	20 (23.5%)	12 (41.4%)	1 (100%)	83 (37.2%)	

**Table 3:** Association between autopsy and religion combined.

	Christian (N = 273)	Other Religion (N = 11)	Unknown Religion (N = 60)	*p*-Value
Autopsy				
No	178 (65.2%)	11 (100%)	39 (65.0%)	0.0395
Yes	95 (34.9%)	0 (0%)	21 (35.0%)	

**Table 4:** Association between autopsy and intensity of treatment in the final day of life.

	No (N = 228)	Yes (N = 116)	*p*-Value *
Curative-Directed Chemotherapy			
Received	19 (8.3%)	14 (12.1%)	0.358
Did not receive	209 (91.7%)	102 (87.9%)	
Medically Administered Nutrition			
Received	176 (77.2%)	90 (77.6%)	1
Did not receive	52 (22.8%)	26 (22.4%)	
Dialysis			
Received	14 (6.1%)	23 (19.8%)	<0.001
Did not receive	214 (93.9%)	93 (80.2%)	
Ventilator			
Received	57 (25.0%)	61 (52.6%)	<0.001
Did not receive	171 (75.0%)	55 (47.4%)	
ICU Location			
No	127 (55.7%)	41 (35.3%)	<0.001
Yes	101 (44.3%)	75 (64.7%)	

* All association tests shown in this table were performed using the Chi-squared test. Adjusting for age and primary diagnosis using logistic regression did not change the significance result (minor *p*-value differences are observed) for these tests.

## Data Availability

Data available upon request from first or last author.
